# Investigation of the Association between the Energy Metabolism of the Insect Vector *Laodelphax striatellus* and Rice Stripe Virus (RSV)

**DOI:** 10.3390/v14102298

**Published:** 2022-10-19

**Authors:** Lu Zhang, Xinyi Li, Yan Chen, Lin Kang, Jiao Zhang, Yao Li, Fang Liu

**Affiliations:** 1 College of Horticulture and Plant Protection, Yangzhou University, Yangzhou 225009, China; 2 Jiangsu Co-Innovation Center for Modern Production Technology of Grain Crops, Yangzhou University, Yangzhou 225009, China

**Keywords:** RSV, *Laodelphax striatellus*, energy metabolism, ATP synthase, mitochondrial import inner membrane translocases, NAD-dependent malic enzyme

## Abstract

Viruses, as intracellular parasites, rely on the host organism to complete their life cycle. Although over 70% of plant viruses are transmitted by insect vectors, the role of vector energy metabolism on the infection process of insect-borne plant viruses is unclear. In this study, full-length cDNAs of three energy metabolism-related genes (*LsATPase*, *LsMIT13* and *LsNADP-ME*) were obtained from the small brown planthopper (SBPH, *Laodelphax striatellus*), which transmits the *Rice stripe virus* (RSV). Expression levels of *LsATPase*, *LsMIT13* and *LsNADP-ME* increased by 105%, 1120% and 259%, respectively, due to RSV infection. The repression of *LsATPase*, *LsMIT13* or *LsNADP-ME* by RNAi had no effect on RSV nucleocapsid protein (NP) transcripts or protein levels. The repression of *LsATPase* caused a significant increase in *LsMIT13* and *LsNADP-ME* transcript levels by 230% and 217%, respectively, and the repression of *LsMIT13* caused a significant increase in *LsNADP-ME* mRNA levels. These results suggested that the silencing of *LsATPase* induced compensatory upregulation of *LsMIT13* and *LsNADP-ME*, and silencing *LsMIT13* induced compensatory upregulation of *LsNADP-ME*. Further study indicated that the co-silencing of *LsATPase*, *LsMIT13* and *LsNADP-ME* in viruliferous SBPHs increased ATP production and RSV loads by 182% and 117%, respectively, as compared with nonviruliferous SBPHs. These findings indicate that SBPH energy metabolism is involved in RSV infection and provide insight into the association between plant viruses and energy metabolism in the insect vector.

## 1. Introduction

Viruses utilize the resources, metabolites and energy of host cells to complete their own life cycle [1,2,3]. There is growing evidence that energy metabolism participates in viral infection in mammals and plant hosts, and the main energy currency for viruses is adenosine 5′-triphosphate (ATP). In humans, ATP was shown to regulate the maturation and assembly of double-stranded DNA (dsDNA) viruses such as *Herpes simplex virus* and *Adenovirus* [1,4,5]. Host cellular ATP accumulates at the replication site of *Hepatitis C virus* (HCV) and supplies the energy required for viral replication [6]. *Tomato bushy stunt virus* (TBSV) hijacks the host plant glycolytic ATP-generating Pgk1 phosphoglycerate kinase and two ATP-producing glycolytic enzymes to produce ATP for viral replication and assembly [7,8]. Although the majority of plant viruses are transmitted by insect vectors [9], the role of insect energy metabolism in viral infection remains unclear.

*Rice stripe virus* (RSV), a member of the genus *Tenuivirus*, is transmitted by the small brown planthopper (SBPH), *Laodelphax striatellus,* in a persistent-propagative manner [10]. It is one of the most destructive rice viruses and significantly reduces rice production [11]. Insect vectors have critical roles in the infection cycle of most plant viruses [12,13]. A recent study demonstrated that 12 ATP-related proteins may interact with the RSV nucleocapsid protein (NP) [14]; however, the role of SBPH energy metabolism in RSV infection is unclear.

Our prior research identified three up-regulated expressed genes encoding ATP synthase (LsATPase), mitochondrial import inner membrane translocase (LsMIT13) and NAD-dependent malic enzyme (LsNADP) in viruliferous SBPH as compared to nonviruliferous individuals [15]. ATP synthase is essential for ATP synthesis via oxidative phosphorylation [16,17]. Mitochondrial import inner membrane translocases participate in the translocation of proteins across the mitochondrial inner membrane [18] and involve coupling of the respiratory chain complex [19]; the latter is comprised of enzymes that create the electrochemical gradient of protons (H+), which is required for the synthesis of ATP [20]. NAD-dependent malic enzyme (NADP-ME) is involved in the citric acid cycle and glycolytic pathway; it is crucial for energy metabolism and preservation of the intracellular redox state [21].

In this study, we cloned *LsATPase*, *LsMIT13* and *LsNADP-ME* from SBPH and analyzed their expression in SBPH. The co-silencing of *LsATPase*, *LsMIT13* and *LsNADP-ME* stimulated energy compensation and facilitated RSV accumulation in SBPH. These insights provide a better understanding of energy metabolism in plant viruses and their insect vectors.

## 2. Materials and Methods

### 2.1. Insects, Plants and RSV Detection

The original strain of *L. striatellus* was a gift provided by Professor Zhou Yijun’s laboratory at the Jiangsu Academy of Agricultural Sciences. All insects were raised on rice (*Oryza sativa*) cv. Wuyujing 3 in a controlled climate chamber with a 16:8 light/dark photoperiod as described [22]. Viruliferous and nonviruliferous SBPHs were maintained separately in glass beakers. To confirm that SBPHs were viruliferous, a single female insect was allowed to feed alone on rice. Parents and offspring were tested monthly for RSV using Dot-ELISA and RSV-specific monoclonal antibodies [22], and highly viruliferous SPBH were collected and used in experiments.

### 2.2. PCR, RACE and Real-Time qRT-PCR

SBPH adults (*n* = 5) were collected as a single sample, and total RNA was extracted with TRIzol as recommended by the manufacturer (cat. 5346994, Invitrogen, Carlsbad, CA, USA). Primers were designed based on available sequences (Table S1), and PCR was conducted with 2 × Phanta Max Master Mix (Vazyme, Nanjing, Jiangsu, China). 5′- and 3′-RACE were conducted to obtain full-length cDNAs of *LsATPase, LsMIT13* and *LsNADP-ME* with the SMART RACE cDNA Amplification Kit as recommended by the manufacturer (Clontech, Mountain View, CA, USA).

Gene-specific primers (Table S1) and the Hieff^®^ qPCR SYBR Green Master Mix (Yeasen, Shanghai, China) were used to conduct real-time qRT-PCR with the Bio-Rad CFX 96 Real-Time PCR system. qRT-PCR conditions were set as follows: 95 °C for 30 s followed by 40 cycles at 95 °C for 10 s and 55 °C for 15 s; a dissociation protocol was then conducted with a gradient from 57 °C to 95 ℃ to verify the amplification specificity and confirm the absence of primer dimers. Expression levels were normalized with *LsActin* using the 2^−ΔΔCT^ method and Bio-Rad CFX Manager v. 2.1 software (Hercules, CA, USA). The means and standard errors were calculated from three or more biologically independent sample sets.

### 2.3. Antibodies

Polyclonal antiserum for RSV NP was raised in mouse and was provided by HuaAn Biotechnology Co., Ltd. (Hangzhou, Zhejiang, China). The following antibodies were obtained from the indicated sources: goat anti-mouse IgG with horseradish peroxidase (HRP) conjugate (Cat. No. CW0102S, Cwbiotech, Taizhou, Jiangsu, China), goat anti-rabbit IgG with HRP conjugate (Cat. CW0103S, Cwbiotech, Taizhou, Jiangsu, China), Alexa Fluor 488-labeled goat anti-mouse IgG (Cat. 115-545-003, Jackson ImmunoResearch Laboratories, West Grove, PA, USA), Alexa Fluor 555-labeled donkey anti-rabbit IgG (Cat. Ab150074, Abcam, Cambridge, MA, UK), rabbit polyclonal anti-GAPDH (Cat. Ab157156, Abcam, Cambridge, MA, UK) and rabbit polyclonal anti-His tag (cat. 2365, Cell Signaling Technology, Danvers, MA, USA).

### 2.4. Immunofluorescence Microscopy

Dissected midgut, ovary and salivary glands were fixed in 4% paraformaldehyde for 1 h, washed three times in PBS (pH 7.4, 10 mM), permeabilized with 1% Triton X-100 in phosphate-buffered saline (PBS) (*v*/*v*) for 1 h, and blocked as described previously [15]. After incubating samples 24 h at 4 °C with RSV NP antibodies, samples were washed in PBS and incubated for 1 h with Alexa Fluor 488 or 555 secondary antibodies at a 1:1000 dilution in PBS (Invitrogen, Carlsbad, CA, USA). Samples were placed on slides in 4′,6-diamidino-2-phenylindole (DAPI) mounting medium (Solarbio, Beijing, China); after a brief rinse in PBS, images were acquired and processed with a Leica TCS SP8 STED 3X high resolution microscope (Leica, Wetzlar, HE, Germany).

### 2.5. RNA Interference (RNAi)

The Transcript Aid™ T7 High Yield Transcription Kit (Thermo Fisher Scientific, Waltham, MA, USA) was used to synthesize dsRNAs using coding sequences of *LsATPase*, *LsMIT13* and *LsNADP-ME*. Approximately 36.8 ng of dsRNAs were injected into 3rd instar nymphs of viruliferous SBPHs with a Nanoliter 2010 injector system (WPI, Sarasota, FL, USA). Insects were harvested on the third day post-dsRNA injection and analyzed for *LsATPase*, *LsMIT13* and *LsNADP-ME* expression by qRT-PCR; dsGFP-injected larvae were used as controls.

### 2.6. Western Blots and Protein Detection

Proteins were isolated, separated by 12–20% SDS-PAGE and transferred to NC membranes as described previously [15]. Probes included anti-RSV NP (1:1000 dilution) and anti-GAPDH (1:5000 dilution). Proteins were detected with goat anti-rabbit IgG-conjugated HRP and goat anti-mouse IgG-conjugated HRP antisera at a 1:5000 dilution. Western blots were imaged as described previously [15].

### 2.7. ATP Assays

After washing the samples twice with PBS, 200 μL lysis buffer was added and the mixture was disrupted by sonication. The lysate was centrifuged at 12,000× *g* for 5 min at 4 °C. The supernatant was transferred to a new 1.5 mL tube for ATP assays with the ATP detection kit (Cat. No. S0026, Beyotime, Shanghai, China).

### 2.8. Statistical Analyses

Relative accumulation of mRNAs was compared using the one-way ANOVA program of SPSS [23]. All data are presented as means ± SE, and *p* < 0.05 was considered statistically significant.

## 3. Results

### 3.1. cDNA Cloning and Sequence Analysis

**Figure 1:**
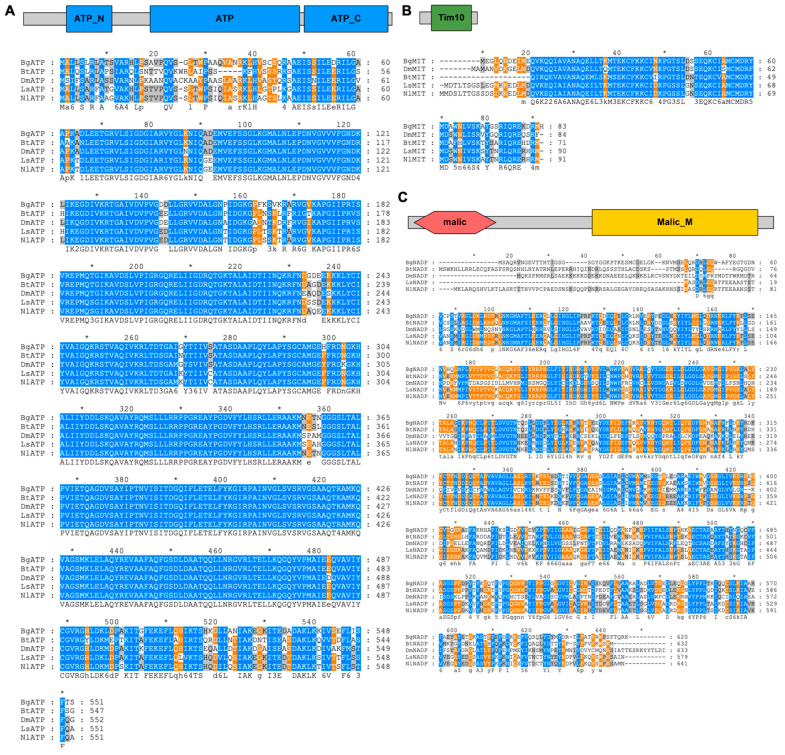
LsATPase, LsMIT13 and LsNADP-ME protein structure and amino acid alignment. Schematic representation and deduced amino acid alignments are shown for (**A**) ATPase, (**B**) MIT13 and (**C**) NADP-ME. The ATP-synthase domains, Tim10_DDP domain, malic domain and malic-M domain are indicated by blue, green, red and yellow boxes, respectively. Abbreviations indicate protein from the following insect species: Ls, *Laodelphax striatellus*; Nl, *Nilaparvata lugens*; Bt, *Bombus terrestris*; Dm, *Drosophila melanogaster*; and Bg, *Blattella germanica*. Alignments were constructed using Clustal W software. Blue shading indicates conserved ATPase residues; orange or grey shading indicates species-specific residues.

Full-length cDNA sequences of *LsATPase*, *LsMIT13* and *LsNADP-ME* were obtained from SBPH adults by RACE-PCR. The 2166 bp full-length cDNA of *LsATPase* (GenBank accession no. KF818471) encoded a predicted protein of 551 amino acids (aa) with three ATP-synthase domains (MW, 59.53 kDa; pI, 9.36) (Figure 1A). The predicted protein sequence for LsATPase had high similarity with Hemipteran ATP synthases (Figure 1A), including NlATPase in *Nilaparvata lugens* (GenBank accession no. XP_022190920.1, 95.64% identity). *LsMIT13* (GenBank accession no. KJ697787) is a 695-bp full-length cDNA encoding a putative protein of 90 aa with a Tim10_DDP domain (MW, 10.40 kDa; pI, 8.32) (Figure 1B). The LsMIT13 sequence shares a high level of identity to other MITs in the Hemiptera (Figure 1B) and shared the greatest similarity with MIT13 from *N. lugens* (NlMIT13, GenBank accession no. XP_022207178.1, 91.21% identity) (Figure 1B). The *LsNADP-ME* (GenBank accession no. KJ697788) is a 2061-bp full-length cDNA encoding a putative protein of 579 amino acids with two malic domains (MW, 64.16 kDa; pI, 8.48) (Figure 1C). LsNADP-ME had high sequence identity to hemipteran NADPs and was closely related to NADP in *N. lugens* (GenBank accession no. XP_022190920.1, 95.75% identity) (Figure 1C).

### 3.2. Expression of LsATPase, LsMIT13 and LsNADP-ME in SBPH

**Figure 2:**
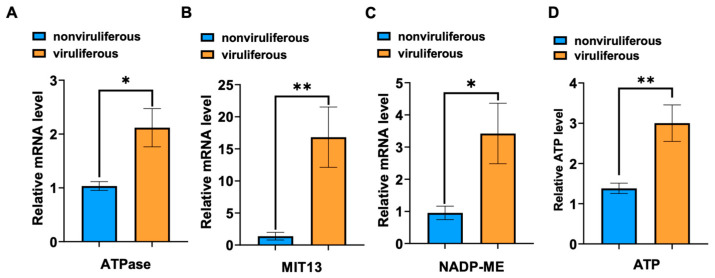
RSV infection increased ATP levels and the expression of *LsATPase*, *LsMIT13* and *LsNADP-ME* in SBPH. Expression levels in viruliferous and nonviruliferous SBPH adults were obtained by RT-qPCR; panels show expression of (**A**) *LsATPase*, (**B**) *LsMIT13* and (**C**) *LsNADP-ME*. Five insects comprised a single replicate, and each treatment contained three replicates. (**D**) ATP levels in viruliferous and nonviruliferous SBPH as determined by ATP bioluminescence assays. Significant differences were obtained using the Student’s *t*-test (* *p* < 0.05 and ** *p* < 0.01; means ± SEM).

To evaluate the effect of RSV infection on the expression of *LsATPase*, *LsMIT13* and *LsNADP-ME*, qRT-PCR was conducted to quantify mRNA levels in 3rd instar nymphs of viruliferous and nonviruliferous SBPHs. RSV infection increased the expression of *LsATPase*, *LsMIT13* and *LsNADP-ME* by 105%, 1120% and 259%, respectively (Figure 2A–C), which indicated that LsATPase, LsMIT13 and LsNADP-ME play important roles in RSV infection and replication in insect vectors. Changes in ATP levels in viruliferous and nonviruliferous SBPHs were monitored by ELISA, and ATP levels in viruliferous SBPH showed a significantly increase (up 117%) as compared with levels in nonviruliferous SBPH (Figure 2D). These results suggest that genes involved in energy metabolism play an important role in RSV infection of SBPH.

### 3.3. RNAi of LsATPase, LsMIT13 or LsNADP-ME has no effect on RSV Loads in SBPHs

**Figure 3:**
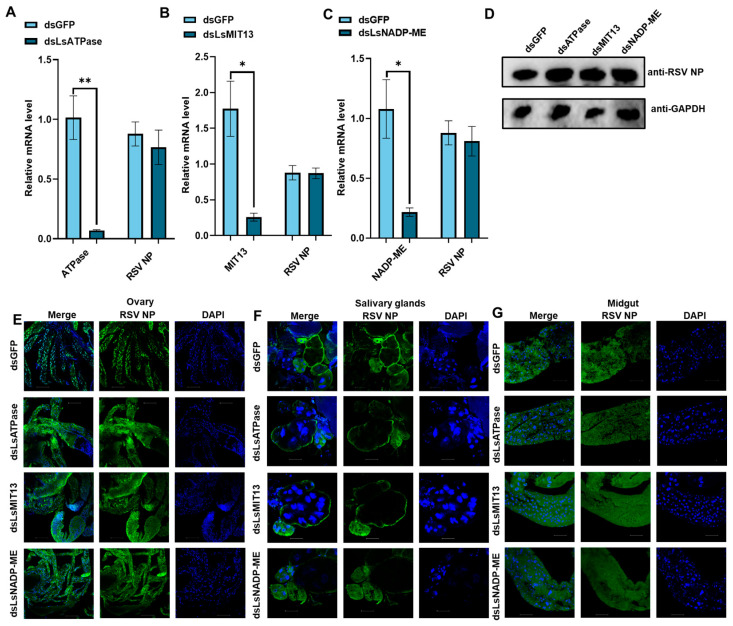
Knockdown of *LsATPase*, *LsMIT13* and *LsNADP-ME* expression and effects on RSV load in SBPH. Panels: (**A**) *LsATPase* expression in dsLsATPase-treated SBPH; (**B**) *LsMIT13* expression in dsLsMIT13-treated SBPH; and (**C**) *LsNADP-ME* expression in dsLsNADP-ME-treated SBPH; dsGFP was used as a control. Each treatment contained three replicates, and significant differences were determined using the Student’s *t*-test (* *p* < 0.05 and ** *p* < 0.01; means ± SEM). (**D**) Western blot analysis of RSV NP in SBPH (*n* = 30) treated with dsLsATPase, dsLsMIT13, dsLsNADP-ME and dsGFP. Antisera for glyceraldehyde-3-phosphate dehydrogenase (GAPDH) was used as control. Immunofluorescence is shown for ovaries (panel **E**), salivary glands (**F**) and midguts (**G**) of viruliferous SBPH treated with dsLsATPase, dsLsMIT13, dsLsNADP-ME and dsGFP. Tissues were immunolabeled with anti-RSV NP (Alexa Fluor 488, green), stained with DAPI (blue) and examined by confocal microscopy. Each treatment was replicated five times. Bar = 50 µm.

The roles of energy metabolism genes in RSV infection were further explored by treating viruliferous nymphs with dsLsATPase, dsLsMIT13, dsLsNADP-ME and dsRNA derived from green fluorescent protein (dsGFP). qRT-PCR analyses showed that treatment with dsATPase, dsLsMIT13 and dsLsNADP-ME significantly reduced *LsATPase* (93%), *LsMIT13* (85%) and *LsNADP-ME* (80%) expression, respectively, as compared to the dsGFP controls (Figure 3A–C). These results indicated that RNAi-mediated knockdown of these three genes was highly effective. Interestingly, treatment of viruliferous SBPH with dsLsATPase, dsLsMIT13 and dsLsNADP-ME did not cause significant changes in RSV NP mRNA and protein levels (Figure 3D). Immunofluorescence assays of RSV NP accumulation indicated that treatment of viruliferous SBPH with dsLsATPase, dsLsMIT13 or dsLsNADP-ME did not affect RSV accumulation in ovaries, salivary glands and midguts (Figure 3E–G).

### 3.4. Co-silencing of LsATPase, LsMIT13 and LsNADP-ME Increases RSV Loads in SBPH

**Figure 4:**
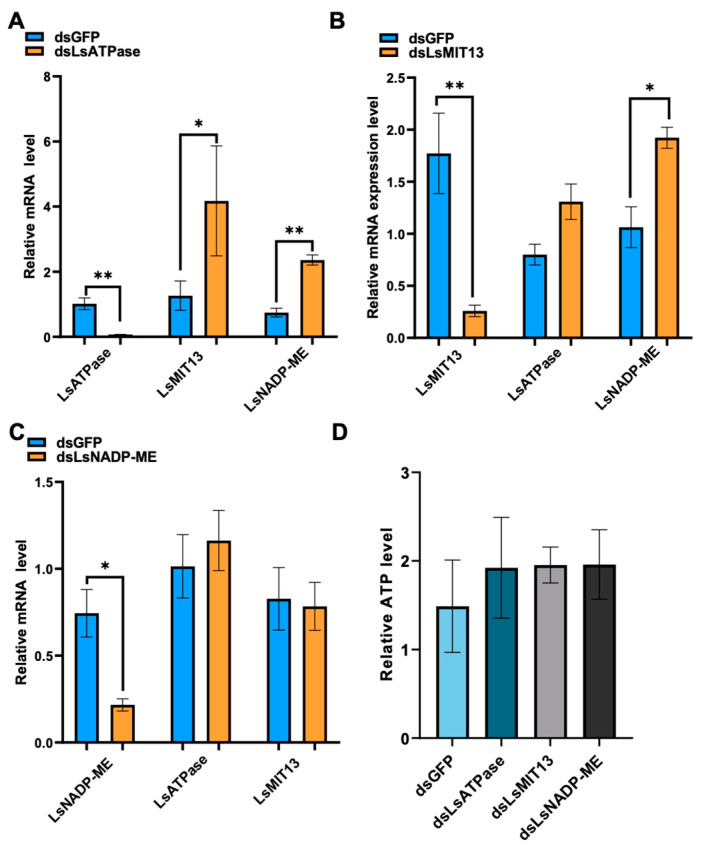
Compensatory regulation of *LsATPase, LsMIT13* and *LsNADP-ME* during RNAi knockdown experiments. Panels show expression levels of *LsATPase*, *LsMIT13* and *LsNADP-ME* in (**A**) dsLsATPase-, (**B**) dsLsMIT13- and (**C**) dsLsNADP-ME-treated SBPHs. Each treatment contained three replicates. (**D**) ATP levels in dsLsATPase-, dsLsMIT13-, dsLsNADP-ME- and dsGFP-treated SBPHs as determined by an ATP bioluminescence assay. Significant differences were evaluated using the Student’s *t*-test (* *p* < 0.05 and ** *p* < 0.01; means ± SEM).

Energy compensation is a common strategy in eukaryotes during adverse conditions for ATP homeostasis [24]. Our results indicated that RSV infection significantly increased the expression of *LsATPase*, *LsMIT13* and *LsNADP-ME*, and the knockdown of *LsATPase*, *LsMIT13* or *LsNADP-ME* had no effect on RSV loads (Figure 3D). This led us to speculate that silencing one of these genes may induce the expression of the other two genes to meet the energy needs within the host or the energy requirements for the virus. To test this hypothesis, we evaluated expression levels of *LsATPase*, *LsMIT13* or *LsNADP-ME* in dsLsATPase-, dsLsMIT13- or dsLsNADP-ME-treated SBPH. qRT-PCR showed that dsLsATPase treatment of viruliferous SBPH increased *LsMIT13* and *LsNADP-ME* expression by 230% and 217%, respectively (Figure 4A). Treatment of SBPH with dsLsMIT13 caused a significant increase (81%) in *LsNADP-ME* expression (Figure 4B), but silencing LsNADP-ME did not impact transcription of *LsATPase* or *LsMIT13* (Figure 4C). We also evaluated ATP levels in dsLsATPase-, dsLsMIT13-, dsLsNADP-ME- and dsGFP-treated viruliferous SBPHs and found no significant difference in ATP content among the four treatments (Figure 4D). These results demonstrated a compensatory relationship between *LsATPase*, *LsMIT13* and *LsNADP-ME*.

**Figure 5:**
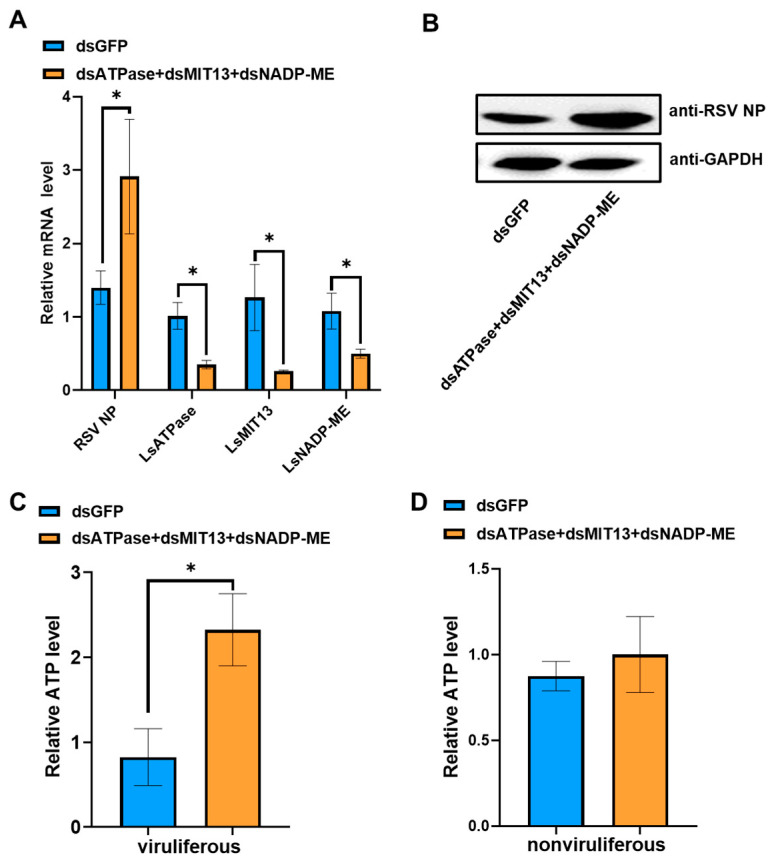
The effects of co-silencing *LsATPase*, *LsMIT13* and *LsNADP-ME* on RSV loads and ATP content. (**A**) qRT-PCR analysis of *LsATPase*, *LsMIT13*, *LsNADP-ME* and RSV NP expression in SBPH co-silenced with dsLsATPase, dsLsMIT13 and dsLsNADP-ME and dsGFP. (**B**) Western blot analysis of RSV NP in co-silenced (dsLsATPase, dsLsMIT13 and dsLsNADP-ME) and dsGFP-treated SBPH. A total of 30 treated SBPH were used for protein extraction. Glyceraldehyde-3-phosphate dehydrogenase (GAPDH) was used as control. (**C**) ATP levels in co-silenced (dsLsATPase, dsLsMIT13 and dsLsNADP-ME) and dsGFP-treated viruliferous SBPH as determined with the ATP bioluminescence assay. (**D**) ATP levels in co-silenced and dsGFP-treated nonviruliferous SBPH as measured with the ATP bioluminescence assay. Significant differences were determined with the Student’s *t*-test (* *p* < 0.05).

To further investigate the roles of *LsATPase*, *LsMIT13* and *LsNADP-ME* on RSV loads, we examined the effect of co-silencing all three genes simultaneously with dsLsATPase, dsLsMIT13 and dsLsNADP-ME. Co-silencing the three genes increased RSV NP mRNA levels (Figure 5A, up 117%) and NP protein levels (Figure 5B). Furthermore, co-silencing *LsATPase*, *LsMIT13* and *LsNADP-ME* increased ATP levels by 182% relative to the dsGFP control in viruliferous SBPH (Figure 5C). ATP levels were not significantly different from the dsGFP control when the three genes were co-silenced in nonviruliferous SBPH (Figure 5D). Thus, the co-silencing of *LsATPase, LsMIT13* and *LsNADP-ME* in viruliferous SBPHs induced higher ATP levels than in nonviruliferous SBPHs, suggesting that RSV infection promotes ATP compensation in SBPH. Overall, our results suggest that the co-silencing of *LsATPase*, *LsMIT13* and *LsNADP-ME* induced more ATP compensation in the presence of RSV, which leads to increased RSV load.

## 4. Discussion

Several studies have shown that the energy metabolism of insect vectors is associated with the plant pathogens they transmit, including plant pathogenic bacteria and microsporidia [25,26]. However, it is unknown the role of insect vectors energy metabolism in viral infection. In this study, we identified three energy metabolism genes in SBPH, namely *LsATPase*, *LsMIT13* and *LsNADP-ME*. ATP levels and transcription of *LsATPase*, *LsMIT13* and *LsNADP-ME* were induced in RSV-infected SBPH. The co-silencing of *LsATPase*, *LsMIT13* and *LsNADP-ME* expression stimulated ATP production and increased RSV loads. These results suggest that SBPH energy metabolism is involved in RSV infection.

Energy compensation is a common strategy that eukaryotes utilize to maintain energy homeostasis during hypoxia and starvation [27,28,29]. For example, cancer cells can deregulate oxidative metabolism and utilize glycolysis to meet energy requirements, which is commonly known as the Warburg effect [29,30]. Fibroblasts of patients with malonyl-CoA synthase deficiencies show increased glycolytic flux, increased lipoylation and impaired mitochondrial respiration, which results in increased β-oxidation and the use of anaplerotic amino acids to supply energy needs [31]. In tumor cells with impaired oxidative phosphorylation, upregulation of the pentose phosphate pathway compensates for reduced energy yield [32]. However, it is unclear the energy compensation mechanism in insects. In this study, silencing *LsATPase* impaired oxidative phosphorylation by upregulating *LsMIT13*, suggesting that compensation occurred in different components of the same SBPH metabolic pathway. Silencing *LsATPase* or *LsMIT13* disrupted oxidative phosphorylation by upregulating *LsNADP-ME*, which is a key gene in the tricarboxylic acid cycle (TCA cycle); this suggests that compensation occurred in different SBPH metabolic pathways. Moreover, co-silencing *LsATPase*, *LsMIT13* and *LsNADP-ME* impaired oxidative phosphorylation and the TCA cycle by stimulating ATP production and increasing RSV loads. Our results suggested that energy metabolism and its compensatory effects play an important role in RSV infection.

Insects have diverse energy metabolism pathways, including fatty acid metabolism, carbohydrate metabolism, the TCA cycle and oxidative phosphorylation [33,34,35]. In this study, we show that the related genes of SBPH oxidative phosphorylation pathway and the TCA cycle may affect RSV infection. Plant carbohydrate metabolism frequently results in the occurrence of starch lesions or ringspots at the sites of viral infection [36,37]. In humans, carbohydrate metabolism is hijacked by the Zika virus in an effort to usurp the generation of cellular energy [38]. Fatty acid metabolism contributes to the replication of multiple human viruses, including the severe acute respiratory syndrome coronavirus 2 (SARS-CoV-2) and hepatitis C virus [39,40,41]. It is unclear whether other SBPH energy metabolism pathways, such as fatty acid and carbohydrate metabolism, are associated with RSV infection.

## Data Availability

Not applicable.

## Supplementary Materials

The following supporting information can be downloaded at: https://www.mdpi.com/article/10.3390/v14102298/s1. Table S1. Oligonucleotide primers.
