# Supplementary material for: Investigation of the Association between the Energy Metabolism of the Insect Vector Laodelphax striatellus and Rice Stripe Virus (RSV)
Source: Viruses. 2022 Oct 19;14(10):2298. doi: 10.3390/v14102298 (PMC9607531; doi:10.3390/v14102298)
Supplement: Supplementary file 1 [file viruses-14-02298-s001.zip › viruses-1962382-supplementary.pdf]

Supplementary Materials:

Table S1. Oligonucleotide primers.

Primer name	Sequence (5′ to 3′)	Description
3′-LsATPase	TACCTGGCTCCCTACTCGGGCTGTG	LsATPase RACE
5′-LsATPase	CAACAGCCTGCTTGGACAAATCG	
3′-LsMIT13	GCCAGGTTCGTCATTAGGAAACTCAG	LsMIT13 RACE
5′-LsMIT13	TCCATATAGCGGTCCATGCACATC	
3′-LsNADP-ME	AACATCAAACCCCATCAGTGCCTACC	LsNADP-ME RACE
5′-LsNADP-ME	GATTGGTAGGCACTGATGGGGTTT	
T7-dsLsATPase-F	TAATACGACTCACTATAGGCACTCACCAGCAAGACCAGA	LsATPase dsRNA
T7-dsLsATPase-R	TAATACGACTCACTATAGGGCAGTAGCGCACACAATGAT	
T7-dsLsMIT13-F	TAATACGACTCACTATAGGATGGATACTCTCACTTCAGG	LsMIT13 dsRNA
T7-dsLsMIT13-R	TAATACGACTCACTATAGGCGGTCCATGCACATCGCAAT	
T7-dsLsNADP-F	TAATACGACTCACTATAGGCTTCTACCGTCGCAAACAG	LsNADP-ME dsRNA
T7-dsLsNADP-R	TAATACGACTCACTATAGGGATACGCTCACCGTCAGTC	
LsATPase-qF	TGGTGGAGTTCTCGTCTGGC	LsATPase qRT-PCR
LsATPase-qR	TGTCGCCCTCCTTGATGTGC	
LsMIT13-qF	CGTGAACGGCATCGTATGTA	LsMIT13 qRT-PCR
LsMIT13-qR	ACCATCTTTTATGCCGTTACTCAC	
LsNADP-ME-qF	GCCAGTGGATCACCGTTTGA	LsNADP-ME qRT-PCR
LsNADP-ME-qR	GCGGCAGTTAGGAAAACGTC	
